# Serial FEM/XFEM-Based Update of Preoperative Brain Images Using Intraoperative MRI

**DOI:** 10.1155/2012/872783

**Published:** 2012-01-12

**Authors:** Lara M. Vigneron, Ludovic Noels, Simon K. Warfield, Jacques G. Verly, Pierre A. Robe

**Affiliations:** ^1^Department of Electrical Engineering and Computer Science, University of Liège, 4000 Liège, Belgium; ^2^Department of Aerospace and Mechanical Engineering, University of Liège, 4000 Liège, Belgium; ^3^Computational Radiology Laboratory, Department of Radiology Children's Hospital Boston, Harvard Medical School, Boston, MA 02115, USA; ^4^Department of Neurosurgery, University of Utrecht Medical Center, 3584 CX Utrecht, The Netherlands

## Abstract

Current neuronavigation systems cannot adapt to changing intraoperative conditions over time. To overcome this limitation, we present an experimental end-to-end system capable of updating 3D preoperative images in the presence of brain shift and successive resections. The heart of our system is a nonrigid registration technique using a biomechanical model, driven by the deformations of key surfaces tracked in successive intraoperative images. The biomechanical model is deformed using FEM or XFEM, depending on the type of deformation under consideration, namely, brain shift or resection. We describe the operation of our system on two patient cases, each comprising five intraoperative MR images, and we demonstrate that our approach significantly improves the alignment of nonrigidly registered images.

## 1. Introduction

Neurosurgery is characterized by the delicate balance between surgical success and potential for devastating side effects. Thanks to multiple technological improvements, the morbidity of neurosurgical interventions has substantially decreased over the last decades, allowing for the resection of previously inoperable lesions. In particular, image-guided neurosurgery (IGNS) devices allow the use of coregistered and fused multimodality 3D images to guide the surgeon's hand and help define preoperatively the boundaries of pathological and predefined functional structures [1]. Meanwhile, new modes of medical imaging have also improved the localization of pathological lesions and their characterization. Medical imaging nowadays includes a wealth of different techniques, such as computed tomography (CT), structural and functional magnetic resonance imaging (sMRI and fMRI), diffusion tensor imaging (DTI), and positron emission tomography (PET). Although the overall accuracy of IGNS is estimated to be 1–2 mm [2], current neuronavigation systems cannot, however, adapt to changing conditions over time. Skull-opening brain shift, brain retraction, cerebrospinal fluid suction, lesion resection, perfusion, and pharmacological manipulation during surgery indeed all alter the 3D morphology of the structures [2–5]. These changes can lead to localization errors that are one order of magnitude larger that IGNS accuracy [1, 2, 6] and may result in incomplete resections or unexpected damage to normal brain. Such inaccuracies could be reduced if one could acquire, throughout surgery, fresh images of the same modalities and quality as the preoperative ones. However, these images are still major challenges. Intraoperative images such as intraoperative MR (iMR) images are—with the exception of a handful surgical facilities—usually acquired using low-field MRI scanners that provide lower resolution and contrast than their preoperative counterparts, and, to this date, several useful imaging modalities, such as PET and possibly MEG, cannot be acquired intraoperatively. One solution is to “bring over” the high-quality preoperative multimodality images into the intraoperative configuration of the brain using a nonrigid registration technique [7–10]. One category of nonrigid registration techniques uses physics-based models, where landmarks are tracked in successive reduced-quality intraoperative images, and their displacement fields drive the deformation of a biomechanical model. The computation is typically based on the finite element method (FEM). So far, most of the mechanical conditions of the brain cannot be estimated in the operating room, such as the volume of cerebrospinal fluid flowing out of the skull cavity, intercellular fluid volume changes that result from mannitol injection, or changes in blood volume and vessel permeability. The fact that an intraoperative image can provide the knowledge of the current state of the brain after some deformation partly eliminates the need for a complete evaluation of these mechanical conditions. The nonrigid registration technique replaces them with the landmark displacements evaluated from successive intraoperative images.

Using a nonrigid registration technique based on a biomechanical model, three types of brain deformations have been identified that require specific modeling, although they depend on common parameters, such as CSF suction, perfusion, or pharmacological manipulation. The first deformation is the brain shift, which appears at the beginning of surgery with the opening of the skull and dura. The suction or leakage of CSF, as well as the release of intracranial pressure caused by tumor growth, generally cause such shift of the brain (note that in this work, we name “brain shift” the specific shift of the brain that occurs after the opening of the skull and dura, before any other surgical act has happened). The brain also shifts with the two other deformations mentioned below. However, for these deformations, we will consider that the shift is a part of these two deformations. The second deformation is the retraction; when target tissues are located deep inside the brain, the surgeon incises brain tissues and inserts a retractor to spread out the tissues, and to create a path towards the target. The third deformation is the resection, that is, the removal, of lesion tissues. Both resection and retraction de facto imply a cut of tissues. In addition, the resection implies that part of tissues is removed. Three deformations can thus be defined in terms of the two elemental actions that change the topology of the brain: the introduction of a discontinuity and the removal of some tissues.

Most studies of brain deformation based on biomechanical models have focused on shifts (the topology of the brain is not modified), that occurs just after the opening of the skull and dura [11–25]. A good review of these different studies can be found in [24, 26–28]. Resection and retraction are more complex to model than (brain) shift. Until recently, their modeling for the specific application of preoperative image update has received much less attention. One of the difficulty for modeling resection and retraction is that both induce a topological change of the brain because some tissue are cut. A method of mesh adaptation [29–31] or remeshing [32–35] must be used in conjunction with FEM if an accurate representation of the location of the cut, for example, the resection cavity or retraction path, is needed to deform the model. Indeed, FEM cannot directly handle discontinuities that go through the FEs, and requires to realign the discontinuity with FE boundaries.

In the field of fracture mechanics, which studies the growth and propagation of cracks in mechanical parts, some methods were developed to avoid using FEM in conjunction with mesh adaptation or remeshing [36]. The extended finite element method (XFEM or X-FEM) appeared in 1999 [37] and has been the object of considerable research since then [38]. XFEM works by allowing the displacement field to be discontinuous within some FEs of the mesh. The mesh does not have to conform to the discontinuities, so that these can be arbitrarily located with respect to the underlying FE mesh. Because XFEM allows an accurate representation of the discontinuities while avoiding mesh adaption or remeshing, and because of the similarity between cracks in mechanical parts and cuts in tissue, we proposed the use of XFEM for handling cut, resection, and retraction in the updating of preoperative images. This paper presents a complete 3D framework for updating multimodal preoperative images with respect to surgical brain deformations, due to brain shift and successive resections, followed and quantified using iMR images. Our approach is modular, and is applied iteratively each time a new intraoperative image is acquired. We take into account successive deformations based on a linear elastic biomechanical model which is deformed using FEM or XFEM, depending on the type of deformation occurring between the pair of iMR images under consideration, namely, brain shift or resection. Some 2D results were presented in [39]. While some 3D results have already been presented for brain shift [40], and initial 3D results for resection [41] modelings, this paper is the first complete and detailed account of the generalization to 3D of our 2D previous work.

The structure of the paper is as follows. In Section 2, we present the state-of-the-art of resection modeling for preoperative image update. In Section 3, we describe our basic strategy for updating preoperative images based on successive intraoperative images. In Section 4, we give detail about our methods and algorithms. In Section 5, we consider two patient cases that illustrate our approach for handling brain shift followed by successive resections. In Section 6, we validate our results. In Section 7, we conclude and discuss future work.

## 2. State-of-the-Art

Among studies that take into account resection for preoperative image update, one should distinguish two categories. The first category of studies models brain deformation using two time-point images, the first image being acquired before surgery has started, the second image being acquired after resection. In this category, the methods that existed for a second image showing some brain shift are adapted for a second image showing some resection. However, the resection is not explicitly modeled. The second category of studies models brain deformation using more than two time-point images, and models at least two successive resections.

Among the first category of studies, Hagemann et al. [42] developed a 2D method for modeling brain deformation between a preoperative MR image and a postoperative MR image, the postoperative image showing a complete resection. The 2D mesh of the biomechanical model corresponded to the underlying pixel grid of the 2D image. The biomechanical model included four different linear elastic laws for the skull/skin region, the whole-brain region, the CSF region, and the image background. They computed the correspondence of the skull boundary, the whole-brain region boundary in the neighborhood of the tumor, and the posterior midline between the two images. They also computed the correspondence between the internal tumor region boundary visible in the preoperative image, and the resection cavity boundary visible in the postoperative image, both boundaries corresponding under the assumption that the resection is complete. The displacements fields of these landmarks drove the deformation of the biomechanical model. As a result, the biomechanical model presented high deformation in the tumor region, which is not physically plausible. However, the resection was complete, and, thus, they were not interested by the displacement field of the biomechanical model in the tumor region itself.

Clatz et al. [12] developed a 3D method for modeling the brain deformation between a preoperative MR image and an iMR image, the latter showing partial or complete resection. The biomechanical model was deformed based on a sparse volume displacement field evaluated from the two images, using a block matching algorithm. In their algorithm, blocks of voxels that presented discriminant structures were selected in the preoperative image. The blocks were then matched to blocks in the iMR image using a similarity criterion, for example, a coefficient of correlation. The value of the similarity criterion was used as a value of confidence in the displacement measured by the block matching algorithm. The biomechanical model was then deformed iteratively, driven by the sparse displacement field of the matched blocks, where a block rejection step was included for measured block displacements initially selected but considered as outliers. In the iMR image, a part, or the totality, of the tumor tissues were resected. The blocks were thus selected and matched in only the healthy-brain region of the two images. They tested their algorithm on six patient cases, and used for validation nine landmarks picked up manually in each image. They found a mean and maximum error on displacements of 0.75 mm and 2.5 mm, respectively. The error increased as one approached the tumor region. They explained this phenomenon by the fact that a substantial number of block matchings were rejected in the tumor neighborhood. The deformation of the biomechanical model in the tumor neighborhood was thus essentially governed by the linear elastic law, and the result might show the limitation of this model. Archip et al. [7] also tested the nonrigid registration method presented in [12] on eleven patient cases, and used the 95% Hausdorff distance [43] for evaluating the alignment of the nonrigidly registered images. As a result, they obtained a mean error of 1.82 mm.

Among the second category of studies, Miga et al. [44] simulated two successive resections. They built a linear poroelastic biomechanical model and preoperatively tagged the tetrahedron FEs that were going to be removed to simulate the brain deformation due to successive resections. The modeling of resection was performed in two steps. First, the preoperatively tagged FEs were removed. This consisted in duplicating the nodes at the boundary of the resection cavity. The nodes were actually not eliminated, which avoids the cost of remeshing operations. Second, a boundary condition was applied to the new boundary of the resection cavity, in order to model the relaxation of strain energy, induced by preoperative tumor growth or surgery acts, stored in the resected tissues, and released after their removal. In this approach, the tissue discontinuity was represented as best as possible with a jagged topology defined by the FE facets defining the boundary of the resection cavity. Forest et al. [45, 46] also modeled the removal of tetrahedra in order to model the action of an ultrasonic aspirator in the context of real-time surgery simulation.

Ferrant et al. [13, 47, 48] modeled successive resections based on several time-point iMR images. Between two successive images, they deformed the biomechanical model, in its current state of update, to take into account the (partial) resections(s) that took place between these two images. The modeling of resection was performed in two steps. First, the biomechanical model, in its current state of update, was deformed in accordance with the displacement field of the healthy-brain boundary between the pair of images under consideration. Second, the FEs that fell into the resection cavity in the second image of the pair were removed, while the FEs that laid across the resection-cavity boundary were cut. To ensure the link between the successive deformed configuration of the biomechanical model, their algorithm kept track of the topology modification between FEs and nodes of the mesh before and after the removal of FEs. They tested their algorithm on one patient case including five iMR images (the first two iMR images being used for brain shift modeling), and used for validation thirty-two landmarks picked up manually in each image. They found a mean and maximum error on the displacements of 0.9 ± 0.7  mm (mean ± standard deviation) and 3.7 mm, respectively. The error increased as one approached the tumor region. They explained this phenomenon by the limited accuracy in the process of picking landmarks in that region, and because the retraction occurring between the second and third images was modeled as a resection, that is, a removal of tissues, even though the tissues were not removed but simply spread out.

The methods described above have been all developed using an FEM-based biomechanical model for intraoperative image registration. Surgical simulation is another research field that broadly uses FEM-based biomechanical model. The objective of a surgical simulator is to provide an interactive manipulation with force feedback of the anatomical part to be operated using various surgical instruments. In order to model a large range surgical procedures, a real-time interactive cutting method should be included in the simulator. Jeřábková and Kuhlen [49] have applied nonlinear XFEM for simulating cut, and have shown that XFEM is successfully efficient for such purpose. 

## 3. Basic Strategy for Serial Preoperative Image Update

**Figure 1:**
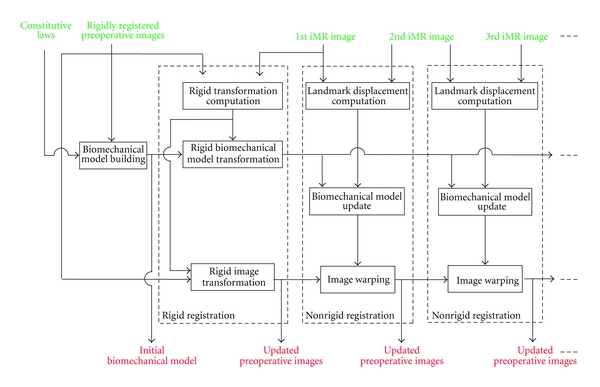
Block diagram of our serial preoperative image-update system dealing with successive brain deformation for a linear formulation.

The block diagram of Figure 1 shows our global approach for updating preoperative images using successive iMR images acquired at different critical points during surgery. Although the principles of the approach are quite general, they are tailored for use based on images acquired with a 0.5 Tesla intraoperative GE Signa scanner, which guarantees that the full volume of brain tissues is included in the image field of view. In our present strategy, the preoperative images are updated incrementally. At the end of each update, the preoperative images should be in the best possible alignment with the last iMR image acquired. The actual algorithms and equations used to this end are described in Section 4.

Prior to surgery, a patient-specific biomechanical model is built from the set of preoperative images. Because the patient does not necessarily lie in the same position during the acquisition of each of the preoperative images, one may need to perform a rigid registration (involving translations, rotations, and scales) to bring these images into correspondence, assuming, in first approximation, there is no local, that is, nonuniform, brain deformation between preoperative images. Once the 1st iMR image has been acquired prior to the opening of the skull, the set of registered preoperative images and the biomechanical model are registered to the 1st iMR image via a rigid transformation. In the present situation, it is assumed that the patient's brain imaged in the 1st iMR image has the same physical shape as the brain imaged in the preoperative images (note that in the following, when an iMR image is defined by a number, this number is the index of the iMR image in the series for a specific patient case. The 1st iMR image thus corresponds to the very first iMR image of the series).

As each iMR image is acquired, this new image and the preceding iMR image are used to estimate the deformation of the brain. The update of the preoperative images is done incrementally with each new pair of successive iMR images. For each pair, we proceed as follows. A set of common anatomical landmarks are tracked between the two iMR images. In our approach, we use as landmarks the surfaces of key brain structures. The use of surface structures rather than volume structures [12] seems more appropriate given the reduced-quality of typical intraoperative images, and would be more easily adapted to intraoperative modalities other than iMR, such as iUS. The landmark surface displacement fields resulting from the matching are then applied to the biomechanical model, which is deformed using FEM or XFEM, depending on the type of deformation occurring between the acquisition times of the iMR images in the pair under consideration, namely, brain shift, or resection. The resulting displacement field of the biomechanical model is finally used to warp the set of preoperative images in their current state of updating. This process is repeated with each new acquisition of an iMR image. Note that, for each deformation modeling, the biomechanical model is deformed in accordance with the landmark displacements tracked between the pair of successive iMR images under consideration. Because intraoperative deformation can follow a reverse direction [5], it is important to track the landmarks between the next-to-last and the last acquired iMR images, rather than track the landmarks between the first and the last acquired iMR images.

For the patient cases treated in Section 5, we assume that the brain undergoes relatively small deformations (small strains and small displacements), and we use a linear finite-element formulation in the biomechanical model. A consequence of using this linear formulation (linear elasticity) is that the equations of solid mechanics can be solved based on the initial configuration of the solid.

Actually, knowing the displacement field increment Δ*u*_*n*_^*n*+1^ = *u* ^*n*+1^ − *u* ^*n*^ at the anatomical landmarks between configuration *n* and increment *n* + 1, one can apply this constrained displacement field increment Δ*u*_*n*_^*n*+1^ to the initial configuration, and the finite element analysis will lead to the deformation tensor increment Δ*ε*_*n*_^*n*+1^ between the configuration *n* and *n* + 1. The final deformation tensor or the body is thus simply obtained from *ε* ^*n*+1^ = ∑_*k*=0_^*n*^ *ε*_*k*_^*k*+1^. Remark that rigorously, the increment of constrained displacement field at the landmark should be applied to the balanced solution of the solid reached after increment *n*, but as we are using a linear elasticity model, this step can be skipped owing to the superposition principle: if *σ* ^*n*+1^ = *Cε* ^*n*+1^, then *σ* ^*n*+1^ = ∑_*k*=0_^*n*^Δ*σ*_*k*_^*k*+1^ = *C*∑_*k*=0_^*n*^ *ε*_*k*_^*k*+1^ = *Cε* ^*n*+1^, where C is the Hooke tensor. As a summary, with this approach, the process of deformations is modeled as a succession of deformations Δ*ε*_*k*_^*k*+1^, for example, brain deformation composed of shift followed by successive resections and the current configuration of the brain biomechanical model, after a specific deformation can then be recovered by adding the computed volume displacements for all successive incremental deformations. Remark that this is not a limitation of the method as we could easily extend it to nonlinear model by simply keeping in memory the previous deformed configuration *n* and adding the constrained displacement field increment Δ*u*_*n*_^*n*+1^ to compute the new deformed configuration at increment *n* + 1, simply this would be less computationally efficient.

Because we use a linear formulation (and, thus, the incremental volume displacement fields can be added to recover the current configuration of the biomechanical model), we could theoretically obtain an identical deformed configuration of the biomechanical model using the two following approaches. The first one would consist of computing and adding the successive incremental deformations of the biomechanical model based on the landmarks tracked between the next-to-last and the last acquired iMR images. The second approach would consist in computing directly the deformed configuration of the biomechanical model based on the landmarks tracked between the first and the last acquired iMR images. However, the landmarks selected to drive the deformation of the biomechanical model vary depending on the type of deformation, namely, brain shift or resection. In addition, part of the biomechanical model is “cut,” using XFEM, to model resection. Consequently, we would not get an identical deformed configuration of the biomechanical model by these two approaches. In order to use a maximum of information from the iMR images, we track, as explained for the first approach, the landmarks between the next-to-last and the last acquired iMR images.

The problem of updating preoperative images between more than two critical points during surgery, that is, based on more than two iMR images, is addressed in only a small number of studies. In our previous work [39, 41], and in [13], the biomechanical model was successively deformed, and this was done using a linear formulation. The framework proposed here, where the initial biomechanical model is always used, instead of using it in its successive states of deformation, has the important advantage of using a good quality mesh for each deformation modeling rather than using a mesh whose quality progressively deteriorates with each successive deformation modeling, and which would require remeshing or mesh adaptation for getting back good FE quality.

To summarize, for each deformation, the landmarks are tracked between the two successive iMR images under consideration. Because we use a linear formulation, the displacement fields of these landmarks are applied to the initial, rather than current, configuration of the biomechanical model. The resulting volume displacement field corresponds to the deformation that the brain undergoes between the two iMR images. This volume displacement field is used to deform the preoperative images in their current state of update, that is, registered (at the previous step, if any) to the first iMR image of the pair. After the deformation, the preoperative images are thus in as good as possible registration to the second iMR image of the pair.

In all the rest of this work, we make a simplification of the approach just presented, by using the 1st iMR image as a substitute for the preoperative images. The biomechanical model is thus built based on structures visible in the 1st iMR image, instead of in the preoperative images, and the structures used in the model are limited to the ones visible in the intraoperative image. Except for the rigid registration between the preoperative images, the biomechanical model, and the 1st iMR image, this simplified approach allows us to discuss, illustrate, and test all key aspects of the system. The 1st iMR image is also updated instead of the preoperative images. The above strategy allows us to focus on the main issue of this paper, that is, the estimation and handling of 3D deformations. Even though the issues involved in the update of preoperative images will need to be addressed in a operational image update system, the present strategy of deforming the iMR images remains useful for calibration purpose, even in the operating room.

## 4. Methods

**Figure 2:**
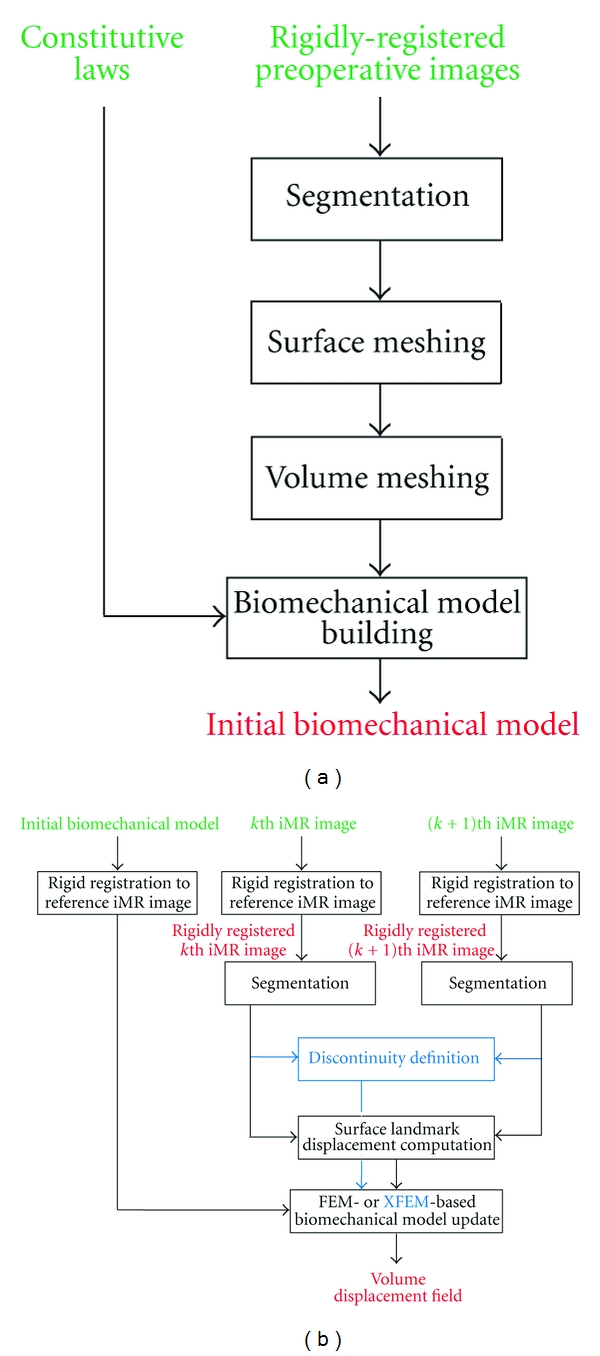
Detailed block diagram of the three subsystems of our serial preoperative image-update system. (a) Building of the biomechanical model from the preoperative images. (b) Calculation of the volume displacement field of the initial biomechanical model using the displacement fields of surface landmarks tracked between a pair of successive iMR images. The updated iMR images are used for validation. For each subsystem, inputs are in green, outputs are in red, and steps related to the definition and use of a discontinuity are in blue.

This section details the different methods that are commonly used for updating preoperative images in presence of brain shift and resection. More specifically, the block diagram of Figure 2(a) shows the building of the biomechanical model from the preoperative images. Specific regions from the preoperative images are segmented, meshed, and assigned appropriate constitutive laws. The block diagram of Figure 2(b) shows, for any pair of successive iMR images, a detailed view of the calculation of the volume displacement field of the initial biomechanical model that corresponds to the deformation that has occurred between the acquisition time of these images.

### 4.1. Rigid Registration of Intraoperative Images

All along surgery, the patient is lying inside the 0.5 Tesla intraoperative GE Signa scanner. Although the patient's head is fixed, one cannot totally rule out the possibility of slight head motion. iMR images thus have to be rigidly coregistered to take into account this potential rigid motion. The rigid registration that we use is the point-based landmark transform available in vtk (http://www.vtk.org/). The corresponding landmark points are manually selected in the successive iMR images.

### 4.2. Segmentation of Intraoperative Images

The segmentation of iMR images into specific regions, for example, healthy-brain and tumor regions, is first performed manually using 3D slicer (http://www.slicer.org/) and then smoothed to minimize the dependance of the results on segmentation roughness. It is clear that performing a manual segmentation in the operating room is not acceptable, and that this process needs to be automated as completely as possible to test the feasibility of our framework online. However, while there exist sophisticated segmentation algorithms that could be used [50–52], in particular for extracting the whole-brain region (skull and external cerebrospinal fluid masked out), the segmentation of the tumor region is still challenging.

### 4.3. Building of Biomechanical Model

As mentioned above, the biomechanical model is built, in the present context, from the 1st iMR image rather than from the preoperative images. Thanks to the use of XFEM instead of FEM for modeling discontinuities, this biomechanical model can be built offline before the operation starts and does not need to be repeated (through remeshing) during the surgery. With respect to FEM-based approaches, the execution time thus ceases to be a limiting parameter, which is a remarkable advantage of our approach. The object to be meshed is defined as a segmented region from an image. It thus requires specific techniques, and we use the meshing software tool isosurf (http://svr-www.eng.cam.ac.uk/~gmt11/software/isosurf/isosurf.html). Our goal is to model the boundaries of healthy-brain and tumor regions as two connected surfaces meshes. However, isosurf can only mesh the boundaries of one or several separate regions, and, thus, does not allow one to mesh connected region boundaries with common nodes at their intersections. We thus start by building two separate surfaces meshes that we connect using our own routines based on vtk. We then smooth the two surface meshes using the software simmetrix (http://www.simmetrix.com/). The two connected triangle surfaces are then jointly meshed into a single volume mesh of tetrahedra that conform to the two surface meshes using gmsh (http://www.geuz.org/gmsh/) [53]. Further details on the building of the biomechanical model, in particular the building of the connected surface meshes, can be found in [40]. A linear elastic law is assigned to the biomechanical model, with Young modulus *E* = 3000 Pa and Poisson ratio *ν* = 0.45 [13]. Because displacements, rather than forces, are applied to the model using a linear formulation, the FEM or XFEM solution is independent of Young modulus *E* [54].

### 4.4. Evaluation of Surface Landmark Displacement Fields

We choose as surface landmarks the whole-brain and internal tumor region boundaries. To evaluate the surface deformations of these region boundaries between two iMR images, we use an active surface algorithm [55, 56]. Because these region boundaries to match must be closed surfaces, we thus use as surface landmarks the whole-brain and healthy-brain region boundaries. The surface deformation of the internal tumor region boundary will be derived from the active surface algorithm of the healthy-brain region boundary. In our active surface algorithm coming from [13, 47, 48], the external forces **F**(**x**) are computed using a gradient descent on a distance map of the region boundary. With such external forces, the active surface algorithm is not able to take correctly into account local rigid motion due, as an example, to lateral or tangential movement depending on the head orientation. For the whole-brain region, any rigid transformation that could have occurred has already been taken into account by the rigid registration of the iMR images (Section 4.1). However, for the healthy-brain region, it can happen that the internal tumor region boundary moves partly in a rigid way. Therefore, the active surface, initialized from the healthy-brain region boundary in the first iMR image, is first locally transformed in a rigid way along the internal tumor region boundary using the iterative closest point transform available in vtk. Then, this resulting surface is deformed using the active surface algorithm as explained above. Further details on the local rigid registration of the healthy-brain region boundary can be found in [40]. Before applying the displacements whole-brain and internal tumor region boundaries to the biomechanical model nodes, the two surface displacement fields are smoothed based on a weighted-distance average, that is, the displacement of each node is averaged with the displacements of its *N* closest neighbor nodes. This smoothing will make them consistent with each other, and compatible with the volume mesh in order to avoid element flipping, in particular at the intersections between whole-brain and internal tumor region boundaries. Depending on the brain deformation modeling, five to ten neighbor nodes are used.

### 4.5. FEM- or XFEM-Based Biomechanical Model Deformation

The displacement fields of the surface landmarks are applied to the biomechanical model, which deforms according to the laws of solid mechanics. The equations of solid mechanics are solved using FEM or XFEM, depending upon the type of circumstances, namely, brain shift or resection. We use the FEM-software tool metafor (http://metafor.ltas.ulg.ac.be/) developed in our mechanical-engineering department, to which we have added an XFEM module. The initial stress state of the brain is unknown and is thus set to zero for each FEM or XFEM computation, as in [10, 13].

FEM discretizes the solid of interest into a mesh, that is, into a set of FEs interconnected by nodes, and approximates the displacement field **u**(**x**) by the FEM displacement field **u** ^FEM^(**x**) defined as

(1) $$\begin{array}{c} u^{\text{FEM}}\left(x\right)=\underset{i\in I}{\sum }\varphi_{i}\left(x\right)u_{i}, \end{array}$$

where *I* is the set of nodes, the *φ*_*i*_(**x**)'s are the nodal shape functions (NSFs), and the **u**_*i*_'s are the nodal degrees of freedom (DOFs). Each *φ*_*i*_(**x**) is defined as being continuous on its compact support **ω**_*i*_, which corresponds to the union of the domains of the FEs connected to node *i* [57]. In our approach, we use linear NSFs.

FEM requires its displacement field **u** ^FEM^(**x**) to be continuous over each FE. In contrast, XFEM handles a discontinuity by allowing the displacement field to be discontinuous within FEs [37, 58–60]. Arbitrarily-shaped discontinuities can then be modeled without any remeshing. The XFEM displacement field generalises the FEM displacement field (1) with

(2) $$\begin{array}{c} u^{\text{XFEM}}\left(x\right)=\underset{i\in I}{\sum }\varphi_{i}\left(x\right)u_{i}+\underset{i\in J}{\sum }\varphi_{i}\left(x\right)\overset{n^{E_{i}}}{\underset{j=1}{\sum }}g_{j}\left(x\right)a_{ji}. \end{array}$$

The first term corresponds to the FEM displacement field (1), where *I* is the set of nodes, the *φ*_*i*_(**x**)'s are the FEM NSFs, and the **u**_*i*_'s are the nodal FEM DOFs. The heart of XFEM is the “enrichment” that adds a number, *n* ^*E*_*i*_^, of DOFs **a**_*ji*_ to each node *i* of the set *J*, which is the subset of nodes of *I* whose support is intersected by the discontinuity of interest. These DOFs are multiplied by the NSFs *φ*_*i*_(**x**) and the discontinuous functions *g*_*j*_(**x**).

The use of specific XFEM enrichment functions *g*_*j*_(**x**) for a node *i* ∈ *J* depends on the type of discontinuity, for example, crack, hole, material interface, and so forth, to be modeled. Suppose that our goal is to model a crack, characterized by a discontinuity in the displacement field (as opposed to a material interface for instance, characterized by a discontinuity in the derivative of the displacement field). When the crack fully intersects the support of the node, a simple choice is a piecewise-constant unit function that changes sign at the boundary across the crack, that is, the Heaviside function

(3) $$\begin{array}{c} H\left(x\right)=\{\begin{array}{cc} 1 & \text{for}\;\left(x-x^{*}\right)\cdot e_{n}>0,\\ -1 & \text{for}\;\left(x-x^{*}\right)\cdot e_{n}<0, \end{array} \end{array}$$

where **x** is again the position of a point of the solid, x* is the position of the point on the crack that is the closest to **x**, and **e**_*n*_ is the outward normal to the crack at x* [37]. In case of resection deformation, the goal is to model a discontinuity such that the part of tissues corresponding to tissue removed by the resection has no influence on the deformation of the remaining part of the tissues. One is actually interested in the deformation of the remaining part of the tissues only. In that sense, the hole function [61] as the following equation:

(4) $$\begin{array}{c} V\left(x\right)=\{\begin{array}{cc} 1 & \text{for}\;\left(x-x^{*}\right)\cdot e_{n}>0,\\ 0 & \text{for}\;\left(x-x^{*}\right)\cdot e_{n}<0, \end{array} \end{array}$$

could be used as XFEM enrichment function, instead of the Heaviside function, and would be totally sufficient. The results that we would obtain on the remaining part of the tissues would be identical. However, because the Heaviside function is necessary for retraction modeling, we have used the same function for the resection modeling even if it was not strictly necessary.

When minimizing the total deformation energy, the resulting XFEM equations remain sparse and symmetric as for FEM. Whereas FEM requires a remeshing and the duplication of the nodes along the crack to take into account any discontinuity, XFEM requires the identification of the nodes whose support is intersected by the crack and the addition of DOFs: (1) any node whose support is not intersected by the discontinuity remains unaffected and thus possesses three DOFs; (2) any node whose support is fully intersected by the discontinuity is enriched with three Heaviside DOFs and thus possesses six DOFs.

### 4.6. Evaluation of Deformation Modeling

To qualitatively estimate the similarity between two images, we compare the edges extracted from these images using the Canny edge detector available in itk (http://www.itk.org/). Indeed, although potentially useful for the sake of comparing methods on a mathematical basis and defining unique correspondences, landmark-based target analysis presents several relevant limitations in the present setting.

- Having experts picking landmarks introduces significant intra- and interobserver variability.
- Picking landmark points, as Ferrant et al. [13] did, is rather difficult when it comes to define enough visible landmarks—especially in the tumor region—on the 5 different images (and not 2 images only, as majority of studies focusing on brain shift are using).
- Rather than point targets, linear tumor contours, and limits between structures and potential eloquent structures matter most in the practical case of tumor ablation neurosurgery.

These are the reason why we chose to use the canny edges in order to evaluate the registration. Besides, while it is true that these edges do not necessarily physically correspond between the successive iMR images, these images have been acquired with the same image protocol (MR sequence, voxel size, grayscale value range), which should limit this problem.

To quantitatively estimate the similarity of the two edge maps, we compute the modified Hausdorff distance between the sets of edge points, that is, voxels representing the edges, in these two images. The modified Hausdorff distance *ℋ*(*A*, *B*) [43] between two sets of points *A* and *B* is defined as

(5) $$\begin{array}{cc} ℋ\left(A,B\right) & =\max\left(h\left(A,B\right),h\left(B,A\right)\right)\;\;\text{with}\;\\ h\left(A,B\right) & =\frac{1}{N_{a}}\underset{a\in A}{\sum }d\left(a,B\right), \end{array}$$

where the directed Hausdorff distance *h*(*A*, *B*) is a measure of the distance of the point set *A* to the point set *B*, *N*_*a*_ is the number of points in set *A*, and *d*(*a*, *B*) is the distance of point *a* ∈ *A* to the closest point in *B*, that is, *d*(*a*, *B*) = min⁡_*b*∈*B*_||*a* − *b*||, where ||*a* − *b*|| is the Euclidean distance. The directed Hausdorff distance *h*(*A*, *B*) thus computes the average distance of points of *A* to points of *B*. The averaging minimizes the effects of outlier points, for example, due to image noise. The value of the modified Hausdorff distance *ℋ*(*A*, *B*) increases with the amount of difference between the two sets of edges points. In the following, we denote by *ℋ*(*I*_*a*_, *I*_*b*_) the modified Hausdorff distance of the edges extracted from the whole-brain region of the images *I*_*a*_ and *I*_*b*_, that is, with the skull and external cerebrospinal fluid masked out from them.

## 5. Results

In this section, we apply our methods, respectively, of brain shift and resection (iMR images are acquired with the 0.5 Tesla intraoperative GE Signa scanner of the Brigham and Women's Hospital, Boston, USA. iMR image size is 256 × 256 × 60 voxels, and voxel size is 0.9375 × 0.9375 × 2.5 mm). All computations are done off-line. Two patient cases, each including five iMR images, are treated to illustrate our modeling and brain shift followed by successive resections. In both cases, the 1st iMR image was acquired prior to the opening of the skull; the 2nd iMR image was acquired after the opening of the skull and dura, and shows some brain shift; the 3rd, 4th, and 5th iMR images were acquired after successive resections. The modelings of brain shift, 1st, 2nd, and 3rd resection are performed using different techniques, as detailed below. Except where otherwise noted, the following discussion applies to both patient cases (the result of each deformation modeling is shown for the two patient cases at the end of Section 5.2.3).

### 5.1. Modeling of Brain Shift

**Figure 3:**
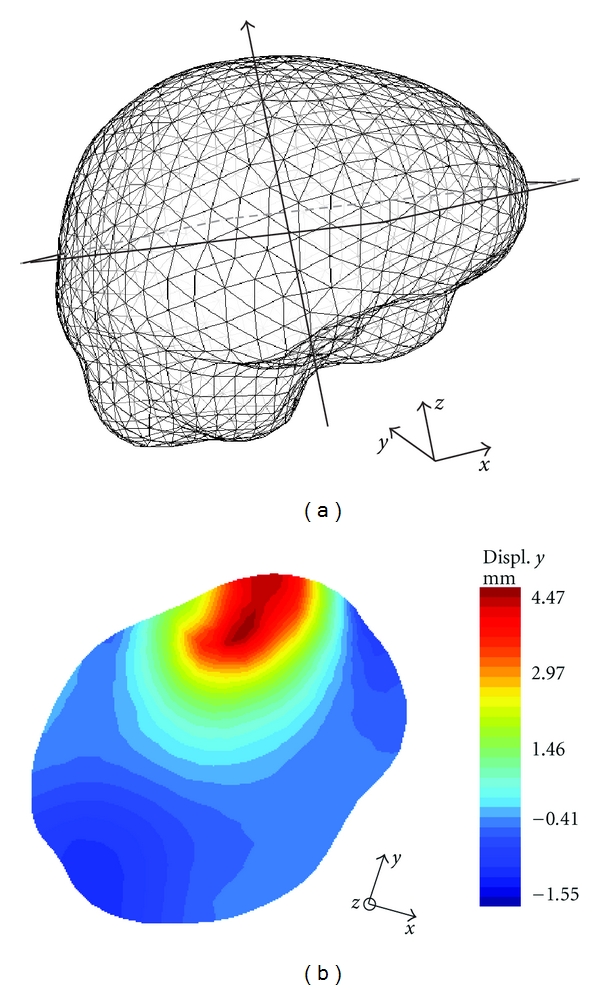
Result of the biomechanical model deformation for brain shift modeling (first patient case). (a) External surface mesh of the biomechanical model with the location of the slice considered in (b). (b) Selected slice of the biomechanical model with color levels corresponding to the displacements along the *y*-axis, which is the main direction of the brain shift for this patient case.

To model brain shift based on the 1st and 2nd iMR images, we estimate the surface displacement fields of the whole-brain region boundary and the internal tumor region boundary from the two iMR images. No tissue discontinuity is involved in the brain shift deformation, so the biomechanical model is deformed using FEM. This results in the volume displacement field of the biomechanical model, which is illustrated in Figure 3 for the first patient case. This volume displacement field is used to warp the part of the 1st iMR image corresponding to the whole-brain region.

### 5.2. Modeling of Successive Resections

In the following sections, the three successive resections are modeled separately, because they require different types of processing. Nevertheless, a common remark can be made for each resection modeling. Matching two region boundaries to get a displacement field makes sense only if they correspond to the same physical entity. Once the resection has started, we can no longer rely on the entirety of the whole-brain region boundary, since a part of it is now missing. For modeling the successive resections, we thus evaluate the displacement field for the boundary of the healthy-brain region only.

#### 5.2.1. Modeling of 1st Resection

The 1st resection occurs between the times the 2nd and 3rd iMR images are acquired. However, since the corresponding removal of tissues is most likely accompanied by deformation, one cannot exactly determine what tissue is removed based just on the two iMR images. We thus decided to model the 1st resection by still relying on the displacement fields of key surfaces, here the healthy-brain region boundary, to deform the biomechanical model. This indeed appears to be the only reliable information concerning the deformation due to resection that we can extract from the 2nd and 3rd iMR images. Consequently, we do not model explicitly the removal of tissue, but we model directly the deformation resulting from it, without introducing any tissue discontinuity. Using the surface displacement field of the healthy-brain region boundary, we compute the deformation of the biomechanical model via FEM. Then, using the resulting volume displacement field, we warp the part of the 2nd iMR image corresponding to the whole-brain region, in the same way as we did in the case of for brain shift. The image resulting from the 1st resection modeling is now registered to the 3rd iMR image, except outside of the healthy-brain region boundary, that is, for the tumor region. Finally, we alter the resulting image to reflect the effect of resection. For this, we assign the background color to the voxels corresponding to the resected tissue volume “absent” in the 3rd iMR image.

#### 5.2.2. Modeling of 2nd Resection

**Figure 4:**
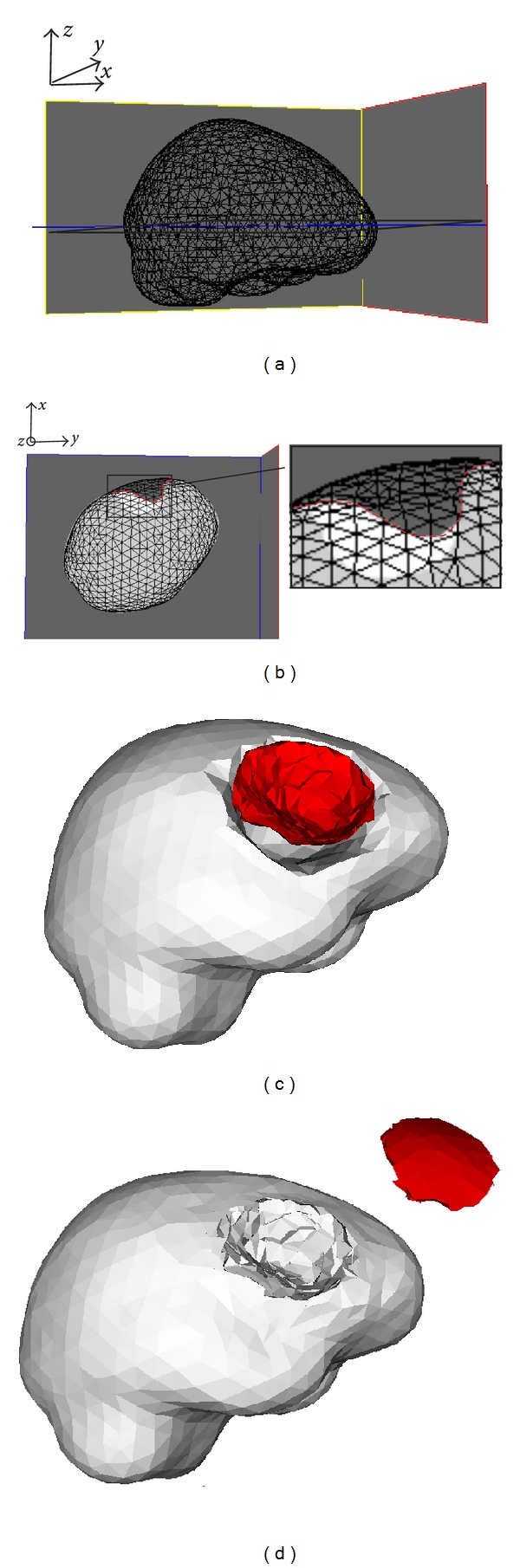
Definition of tissue discontinuity for 2nd resection modeling (first patient case). (a) External surface mesh (of the biomechanical model) with the location of the slice considered in (b). (b) External surface mesh superposed to the healthy-brain region (light gray) and tumor region (white) segmented out from the 3rd iMR image. This superposition allows one to define the tissue discontinuity (red boundary). (c) Surface meshes describing the healthy-brain region boundary (gray) and the tissue discontinuity (red). This tissue discontinuity gives an idea of the part of tumor tissue that was removed by the 1st resection. The gap that appears “between” the gray and red surfaces corresponds to the remaining tumor tissues. (d) Final mesh resulting from the modeling of the 2nd resection using XFEM. The tetrahedra that were added to display separately the two parts of the mesh are only for visualization purposes.

The significant feature of the 2nd resection is that some tissue has already been removed by the 1st resection, which means that this tissue cannot have any physical influence on subsequent brain deformations because it does not “exist” anymore. Consequently, the 1st resection must be reflected in the biomechanical model. Recall that the biomechanical model has been deformed to model the brain shift and the 1st resection and is thus registered to the 3rd iMR image. So, using the 3rd iMR image, we can define the boundary of the 1st resection, that is, the tissue discontinuity to include in the deformed biomechanical model (Figures 4(a) and 4(b)). We then enrich the nodes whose supports are intersected by the discontinuity with Heaviside DOFs. Consequently, when the XFEM-based biomechanical model deforms, the part corresponding to tissue removed by the 1st resection has no influence on the deformation of the remaining part of the brain. For the first patient case illustrated in Figure 4, the tetrahedron mesh consists of 3,317 nodes, which corresponds to 9,951 FEM DOFs. Enrichment adds 873 Heaviside DOFs.

As for the modeling of the 1st resection, the biomechanical model is deformed in accordance with the displacement field of the healthy-brain region boundary evaluated from the 3rd and 4th iMR images.  Figure 4(d) shows the deformed mesh, result of the XFEM computation. The bottom part of the mesh, representing the tissue remaining after the 1st resection, has been deformed according to the displacement field of the healthy-brain region boundary, while the top part, representing the tissue removed by the 1st resection, has been subjected to a translation, but only for visualization purposes. Even though the mesh is displayed as two separate parts, it is, in fact, a single entity. Indeed, a main feature of XFEM is its ability to handle the effect of a discontinuity without modifying the underlying mesh, that is, without remeshing. For modeling the 2nd resection, the edges of FEs straddling the discontinuity have been made discontinuous and their nodes moved apart. Using the XFEM volume displacement field, we warp the part of the 3rd iMR image corresponding to the whole-brain region. The resulting image is then masked out with the whole-brain region segmented out from the 4th iMR image.

#### 5.2.3. Modeling of 3rd Resection

One significant feature of the procedure described for modeling the 2nd resection is that it can be applied repetitively for each subsequent resection visible on successive iMR images, no matter how many there are. The modeling of the 3rd resection is thus identical to the modeling of the 2nd resection. The tissue discontinuity due to the 2nd resection is defined from the 4th iMR image, and used to appropriately enrich the nodes of the biomechanical model. Then, this biomechanical model is deformed using XFEM, in accordance with the displacement field of the healthy-brain region boundary evaluated from the 4th and 5th iMR images.

For the first patient case, a simplification for the modeling of the 3rd resection can be made because, by the time the 5th iMR image is acquired, the resection is complete. This means that we only need to compute the volume displacement field of the healthy-brain region. Since we apply displacements exactly to the boundary of the healthy-brain region, the results obtained with FEM and XFEM will be identical. Using the FEM (for the first patient case) or XFEM (for the second patient case) volume displacement field, we warp the part of the 4th iMR image corresponding to the whole-brain region. The resulting image is then masked out with the whole-brain region segmented out from the 5th iMR image.

**Figure 5:**
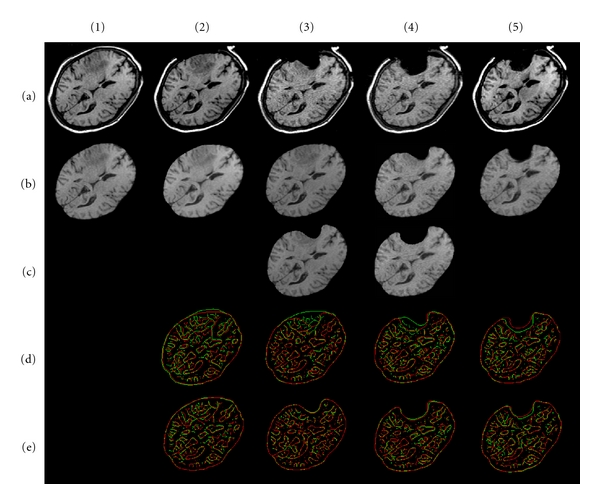
First patient case. (a) Sequence of five input iMR images rigidly registered to the first one. (1b) Whole-brain region extracted from (1a). (2b) Deformation of (1b) computed using FEM for brain shift modeling. (3b) Deformation of whole-brain region extracted from (2a) computed using FEM for 1st resection modeling. (3c) Masking of (3b) with whole-brain region segmented from the 3rd iMR image (3a). (4b) Deformation of whole-brain region extracted from (3a) computed using XFEM for 2nd resection modeling. (4c) Masking of (4b) with whole-brain region segmented from 4th iMR image (4a). (5b) Deformation of whole-brain region extracted from (4a) computed using FEM for 3rd resection modeling. (d) Juxtaposition of Canny edges of images rigidly registered. The edges of the first (second) image of the pair under consideration are in green (red). (e) Ditto for (d) when images are nonrigidly registered.

**Figure 6:**
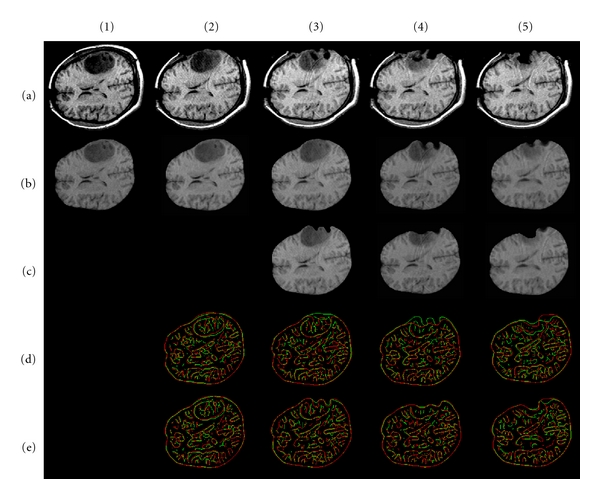
Second patient case. (a) Sequence of five input iMR images rigidly registered to the first one. (1b) Whole-brain region extracted from (1a). (2b) Deformation of (1b) computed using FEM for brain shift modeling. (3b) Deformation of whole-brain region extracted from (2a) computed using FEM for 1st resection modeling. (3c) Masking of (3b) with whole-brain region segmented from the 3rd iMR image (3a). (4b) Deformation of whole-brain region extracted from (3a) computed using XFEM for 2nd resection modeling. (4c) Masking of (4b) with whole-brain region segmented from 4th iMR image (4a). (5b) Deformation of whole-brain region extracted from (4a) computed using XFEM for 3rd resection modeling. (5c) Masking of (5b) with whole-brain region segmented from 5th iMR image (5a). (d) Juxtaposition of Canny edges of images rigidly registered. The edges of the first (second) image of the pair under consideration are in green (red). (e) Ditto for (d) when images are nonrigidly registered.

Figures 5 and 6 show the results of warping the iMR images, as well as the edges extracted from them, after brain shift and each successive resection modeling for the two patient cases.

#### 5.2.4. Comparison of FEM and XFEM for Modeling of Resection

**Figure 7:**
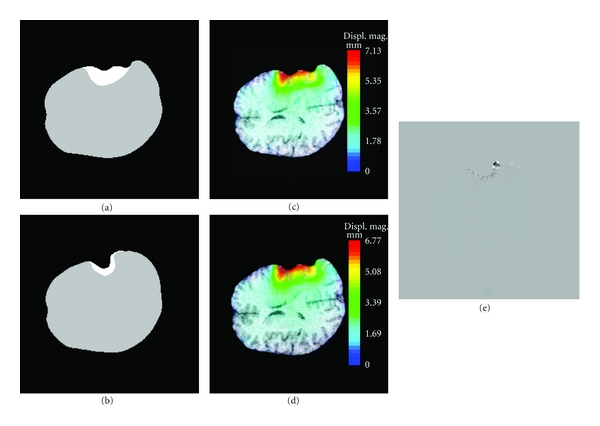
Difference of results using XFEM and FEM for 3rd resection modeling (second patient case). (a) Healthy-brain (gray) and tumor (white) regions segmented out from the 4th iMR image. (b) Healthy-brain and tumor regions segmented out from the 5th iMR image. (c) Volume displacement field of biomechanical model using XFEM. The part of tissue falling within the resection cavity is modeled as being removed. Color levels correspond to the magnitude of the displacement field. (d) Same as (c), but for FEM. The part of tissue falling within the resection cavity is present in the deformation modeling even though it no longer exists. Difference of magnitude between volume displacement fields using XFEM (c) and FEM (d) does not exceed 0.36 mm. (e) Difference in the warping of the part of the 4th iMR image corresponding to the whole-brain region using XFEM and FEM.

As explained in Section 5.2.3, since we apply displacements exactly to the boundary of the healthy-brain region, the results obtained with FEM and XFEM are identical in the healthy-brain region. One can deduce that using XFEM for modeling resection is interesting when the neurosurgeon needs to have an accurate displacement field of the remaining tumor tissues. In this case, it is interesting to evaluate the impact of using FEM, instead of XFEM, to model the resection as if no resection was performed before. Using FEM for modeling resection is equivalent to ignoring the presence of resection on intraoperative images. To illustrate the comparison between FEM and XFEM results, we choose the 3rd resection modeling of the second patient case. Indeed, it is the deformation with remaining tumor tissues that shows the largest magnitude, and, thus, that is likely to give a maximum difference between the two computations.  Figure 7 compares the results obtained using FEM and XFEM. The healthy-brain and tumor regions segmented out from the 4th and 5th iMR images are respectively shown in Figures 7(a) and 7(b). The volume displacement fields of the biomechanical model using XFEM and FEM are respectively shown in Figures 7(c) and 7(d). The part of the 4th iMR image corresponding to the whole-brain region is warped, first with the volume displacement field obtained via FEM, and then with that obtained via XFEM. The difference between the two warped images is shown in Figure 7(e). As expected, there is a visible difference in the remaining tumor tissue. However, the difference between the two volume displacement fields is smaller than the image resolution (although the difference between the two volume displacement fields is smaller than the image resolution, the difference between the images resulting of the warping using these two volume displacement fields is nonzero. This is explained by the fact that the (gray) value of each voxel of the warped image is defined as a weighted-value of voxels of the original image. The weights are defined based on the overlapping ratio of the voxel of the warped image, with voxels (determined using the volume displacement field) of the original image). In addition, the deformed 4th iMR images, using the XFEM- and the FEM-based deformations of the biomechanical model, show the same similarity, computed based on the modified Hausdorff distance, with the 5th iMR.

Two reasons explain that the differences between the FEM and XFEM results are so small. First, the brain deformation itself due to the 3rd resection is small, and, thus, it is expected to obtain small differences between the two resulting brain deformations. Second, in the case the remaining tumor tissues are close to the healthy-brain region boundary, it implies that they are close to the boundary where surface displacement fields are applied to drive the deformation of the biomechanical model. This proximity decreases the influence of the modeling of already resected tissues with XFEM. Although this comparison between FEM and XFEM should be done on more patient cases, we suggest that, in first approximation, FEM could be used for modeling resection cases with small brain deformations. Nevertheless, the presentation of the successive resections using XFEM shows the generality of our framework, and details how XFEM is implemented. Note that in Section 6 devoted to validation, the warped images are the ones deformed with XFEM.

## 6. Validation

For each deformation modeling based on a pair (*I*_*k*_, *I*_*k*+1_) of two successive iMR images that are already rigidly registered, we compare the similarity between these *I*_*k*_ and *I*_*k*+1_ images, as well as the similarity between the *I*_*k*_^*w*^ and *I*_*k*+1_ images, where *I*_*k*_^*w*^ is the result of warping *I*_*k*_. This gives us an estimate of how well we are able to capture, and compensate for, the local deformations between *I*_*k*_ and *I*_*k*+1_. The goal of the nonrigid registration is, however, to deform the preoperative images. By warping *I*_*k*_ for each deformation modeling, we do not take into account the fact that an error of alignment after each deformation modeling could propagate and amplify through the successive deformation modelings. To evaluate the effect of this error amplification on the results, we also perform the required succession of warpings on *I*_1_, and we denote the resulting image by *I*_1,*k*_^*w*^. We then compare, for each deformation modeling, the similarity between *I*_1_ and *I*_*k*+1_, together with the similarity between *I*_1,*k*_^*w*^ and *I*_*k*+1_. This allows one to evaluate the propagation, that is, the amplification, of alignment error on the results. The modified Hausdorff distance computed for each pair of iMR images are given in Tables 1 and 2.

Table 1 shows, for each deformation modeling based on a pair (*I*_*k*_, *I*_*k*+1_) of two successive iMR images, the values of the modified Hausdorff distances *ℋ*(*I*_*k*_, *I*_*k*+1_) and *ℋ*(*I*_*k*_^*w*^, *I*_*k*+1_). These values are computed using the Canny edges extracted from the pair of images (*I*_*k*_, *I*_*k*+1_) (Figures 5 (d) and 6 (d)) and (*I*_*k*_^*w*^, *I*_*k*+1_) (Figures 5 (e) and 6 (e)). We observe that the values for the images nonrigidly registered are relatively constant, that is, *∼*1 mm, for each deformation modeling. Six out of eight deformation modelings give smaller modified Hausdorff distances when the iMR images are (rigidly and subsequently) nonrigidly registered. However, the modified Hausdorff distance increases for the 3rd resection modeling of the first patient case, as well as for the brain shift modeling of the second patient case. To understand if the nonrigid registration is responsible for the increase of the misalignment of the two iMR images everywhere in the whole-brain region, or if this effect is localized, we compute the modified Hausdorff distance in the region and neighborhood of the tumor only (volume region that extents by 25 mm the tumor region segmented in *I*_1_ for both patient cases). The modified Hausdorff distance decreases from *ℋ*(*I*_4_, *I*_5_) = 1.70 mm to *ℋ*(*I*_4_^*w*^, *I*_5_) = 1.37 mm for the first patient case, while it decreases from *ℋ*(*I*_1_, *I*_2_) = 1.36 mm to *ℋ*(*I*_1_^*w*^, *I*_2_) = 1.28 mm for the second patient case. This indicates that the nonrigid registration enhances the alignment of the two iMR images within the tumor region and its neighborhood, which is in fact the location requiring the best modeling accuracy. This behavior could be explained by the fact that a maximum of information from the iMR images is used in this region, that is, one or two (in case of brain shift modeling) surface displacement fields are applied around it. The increase of misalignment elsewhere in the brain volume could be explained by two reasons. First, the landmarks tracked from the iMR images are surfaces. As a consequence, the nonrigid registration is expected to give better results near the tracked surfaces than far from them in the volume [13]. Second, the volume displacement field strongly depends on the constitutive laws. The volume misalignment could point out the need for better parameters values and/or other constitutive laws.

Table 2 shows, for each deformation modeling based on a pair (*I*_*k*_, *I*_*k*+1_) of two successive iMR images, the values of the modified Hausdorff distances *ℋ*(*I*_1_, *I*_*k*+1_) and *ℋ*(*I*_1,*k*_^*w*^, *I*_*k*+1_). So far, IGNS systems allow one to rigidly register preoperative and successive iMR images. *ℋ*(*I*_1_, *I*_*k*+1_) thus represents the navigation accuracy that we can obtain with an IGNS system at the present time. The comparison of *ℋ*(*I*_1_, *I*_*k*+1_) with *ℋ*(*I*_1,*k*_^*w*^, *I*_*k*+1_) gives the improvement that could be practically achieved in the alignment with our approach. As expected, Table 2 shows that the IGNS accuracy decreases through the successive deformations. Indeed, the modified Hausdorff distance increases from *ℋ*(*I*_1_, *I*_2_) = 1.24 mm to *ℋ*(*I*_1_, *I*_5_) = 1.78 mm for the first patient case, and from *ℋ*(*I*_1_, *I*_2_) = 1.01 mm to *ℋ*(*I*_1_, *I*_5_) = 1.68 mm for the second patient case. Six out of eight deformation modelings give smaller modified Hausdorff distances when the iMR images are nonrigidly registered. To understand if the modified Hausdorff distance increases everywhere in the whole-brain region for the brain shift and 1st resection modeling of the second patient case, we compute the modified Hausdorff distance in the neighborhood of the tumor region (in the same way as explained for Table 1), and observe the improvement of the alignment within the tumor region and its neighborhood. As opposed to the values of the modified Hausdorff distances in Table 1, the values for the images nonrigidly registered in Table 2 increase through the successive resection modeling. This amplification error is due to the fact that, after having modeled brain deformation between a pair of iMR images, the deformed biomechanical model is not in perfect alignment with the second image of the pair. Since, for the subsequent deformation modeling, the surface landmarks are initialized based on the deformed biomechanical model, this can thus ampliy a misregistration error.

## 7. Conclusions and Future Work

We developed a complete 3D framework for serial preoperative image update in the presence of brain shift followed by successive resections. The results were presented for two patient cases, each containing five iMR images. The nonrigid registration technique used an homogeneous linear elastic biomechanical model, driven by the deformations of whole-brain and internal tumor region boundaries for brain shift modeling, and healthy-brain region boundary for resection modelings, tracked between successive iMR images. The biomechanical model was deformed using FEM for brain shift modeling, and FEM or XFEM for resection modeling, depending upon whether some brain tissues were previously resected or not. We showed that our approach was modular, and could be applied each time a new iMR image is acquired.

We used a linear formulation to characterize the deformation of the brains of both patients because the brains underwent relatively small deformations and displacements. While nonlinear biomechanical models have proven effective to decrease—yet do not abolish—the inaccuracies of FEM-based modeling methods of large brain deformations, the deformations observed in our patients during surgery remained moderate (4–7 mm), thus reducing the theoretical benefit of using nonlinear models. This allowed us to use simpler linear models and focus on the added value of XFEM to simultaneously account for surgical deformations, namely, shift and resection. Using a linear formulation implied that, for each new deformation modeling, one could use the initial configuration rather than the last-deformed configuration of the biomechanical model. This had the important advantage of using a good quality mesh for each deformation modeling rather than using a mesh whose quality progressively degraded with each successive deformation modeling. This also had the advantage that we did no longer need to reconnect the deformed mesh for each new XFEM calculation, which was one drawback of our previous method, presented in [39, 41], where the biomechanical model was successively deformed. We also showed how XFEM could handle a discontinuity for modeling resection without any remeshing or mesh adaptation while the representation of the discontinuity remained accurate, that is, the representation of the discontinuity was not based on a jagged topology using FE facets. XFEM thus also avoided making the mesh resolution richer in the neighborhood of the resection-cavity boundary for improving the accuracy of the representation of the discontinuity for that purpose only.

We showed that our nonrigid registration technique improved the alignment of the successive iMR images for most of the deformation modeling of both patient cases. When our nonrigid registration failed, it still improved the alignment locally, that is, within the tumor region and its neighborhood. We tested the explicit modeling of the lateral ventricles' region with a soft, compressible law in addition to the whole-brain region law used in the homogeneous biomechanical model. However, it did not have a significative impact on the result.

In addition to the validation that is usually performed for successive deformation modelings, that is, validation between pairs of successive intraoperative images, shown in Table 1 of Section 6 or in the work of Ferrant et al. [13, 47, 48], we also evaluated the fact that an error of alignment after each deformation modeling could propagate and amplify through the successive deformation modelings. As a result, shown in Table 2 of Section 6, we showed that our approach suffered from the propagation of misregistration through the successive deformation modelings. We expected that this was due, at least partly, to the algorithms used to evaluate intraoperative surface displacements fields from the whole-brain and healthy-brain region boundaries. These boundaries were first manually segmented, and then smoothed. The surface displacement fields were computed using active surface algorithms, and smoothed to make them compatible with the biomechanical model. Because of these two smoothings, the deformed biomechanical model was likely to not be in a perfect alignment with the iMR image to which it was registered. Because the surface displacement fields evaluated for the next deformation modeling were initialized based on the deformed biomechanical model, we expected to observe an amplification of the misregistration, which was confirmed by our quantitative evaluation. At the present time though, commercial IGNS systems allow one to register preoperative images and successive iMR images, but in a rigid way only. Consequently, although the effect of error amplification exists, our technique still enhances the current capabilities of commercial IGNS systems.

Future work on modeling of brain shift followed by successive resection is required in five main areas. First, the effect of error amplification through the successive brain deformation modelings calls for further research. Consequently, the segmentation, and the subsequent smoothing, as well as the evaluation of surface displacement fields, should be improved to minimize the effect of error amplification. Second, further research is required to include additional structures in the biomechanical model in general, and to study the best way to include the lateral ventricles in particular. The use of a poroelastic model in order to model the cerebrospinal fluid filling the ventricles could be considered [17, 18]. Third, the fact that we use iMR images could be further exploited. Indeed, these images provide volume information (rather than surface information only), are of good quality in comparison to other intraoperative modalities, and possess a field of view that includes the full volume of brain tissues (for the 0.5 Tesla GE Signa scanner). These images thus allow one to evaluate what, and how, new structures of the brain could be used, to enhance the modeling of brain shift. Some regions, for example, the lateral ventricles' region, could be extracted from the two iMR images, and used as surface landmarks to drive the deformation of the biomechanical model [13, 62]. Indeed, the workflow presented in this paper has the advantage of being easily adaptable. In case the tumor region would not be visible (enough) on the iMR images, these new structures, easier to segment, could also adequately replace the tumor for driving the deformation. Fourth, our global approach should no longer be based on the 1st iMR image used as a substitute for preoperative images, but on the preoperative images themselves. Fifth, we should implement, for the surgery cases involving large deformations of the brain, a nonlinear formulation of FEM [63, 64], and, particularly, a nonlinear formulation of XFEM, which is the subject of recent research [65, 66].

## Figures and Tables

**Table 1:** Values of *ℋ*(*I*_*k*_, *I*_*k*+1_) and *ℋ*(*I*_*k*_^*w*^, *I*_*k*+1_), *k* = 1,…, 4, for each deformation modeling based on a pair (*I*_*k*_, *I*_*k*+1_) of two successive iMR images. First value gives measure of similarity of images rigidly registered, while second value gives measure of similarity of images both rigidly, and (subsequently) nonrigidly registered. For each *I*_*k*_, only the whole-brain region is taken into account for edge extraction.

Modified Hausdorff distance (mm) between		Brain shift		1st resection		2nd resection		3rd resection	
edges extracted from two iMR images		*ℋ*(*I*_1_, *I*_2_)	*ℋ*(*I*_1_^*w*^, *I*_2_)	*ℋ*(*I*_2_, *I*_3_)	*ℋ*(*I*_2_^*w*^, *I*_3_)	*ℋ*(*I*_3_, *I*_4_)	*ℋ*(*I*_3_^*w*^, *I*_4_)	*ℋ*(*I*_4_, *I*_5_)	*ℋ*(*I*_4_^*w*^, *I*_5_)
Patient 1	Whole-brain region	1.24	1.07	0.84	0.69	1.10	0.97	0.96	0.97
	Tumor region and neighborhood							1.70	1.37

Patient 2	Whole-brain region	1.01	1.04	1.07	1.04	1.02	0.93	1.23	1.06
	Tumor region and neighborhood	1.36	1.28						

**Table 2:** Values of *ℋ*(*I*_1_, *I*_*k*+1_) and *ℋ*(*I*_1,*k*_^*w*^, *I*_*k*+1_), *k* = 1,…, 4, for each deformation modeling based on a pair (*I*_*k*_, *I*_*k*+1_) of two successive iMR images. In contrast with Table 1, *I*_1_ is successively warped, rather than *I*_*k*_, for each deformation modeling. First value gives measure of similarity of images rigidly registered, while second value gives measure of similarity of images both rigidly and (subsequently) nonrigidly registered. For each *I*_*k*_, only the whole-brain region is taken into account for edge extraction.

Modified Hausdorff distance (mm) between		Brain shift		1st resection		2nd resection		3rd resection	
edges extracted from two iMR images		*ℋ*(*I*_1_, *I*_2_)	*ℋ*(*I*_1_^*w*^, *I*_2_)	*ℋ*(*I*_1_, *I*_3_)	*ℋ*(*I*_1,2_^*w*^, *I*_3_)	*ℋ*(*I*_1_, *I*_4_)	*ℋ*(*I*_1,3_^*w*^, *I*_4_)	*ℋ*(*I*_1_, *I*_5_)	*ℋ*(*I*_1,4_^*w*^, *I*_5_)
Patient 1	Whole-brain region	1.24	1.07	1.50	1.21	1.80	1.31	1.78	1.38
	Tumor region and neighborhood								

Patient 2	Whole-brain region	1.01	1.04	1.10	1.16	1.36	1.31	1.68	1.42
	Tumor region and neighborhood	1.36	1.28	1.76	1.44				
